# Correction: Metabolic Evolution of a Deep-Branching Hyperthermophilic Chemoautotrophic Bacterium

**DOI:** 10.1371/journal.pone.0093345

**Published:** 2014-03-19

**Authors:** The PLOS ONE Staff

In the online version of the article, the affiliations for both authors are incorrect. Both the first author, Rogier Braakman, and the second author, Eric Smith, should be indicated as affiliated with institution number 1, Santa Fe Institute, Santa Fe, New Mexico, United States of America. Only the second author should be affiliated with institution number 2, Krasnow Institute for Advanced Study, George Mason University, Fairfax, Virginia, United States of America.
