# Supplementary material for: Metabolic Evolution of a Deep-Branching Hyperthermophilic Chemoautotrophic Bacterium
Source: PLoS One. 2014 Feb 5;9(2):e87950. doi: 10.1371/journal.pone.0087950 (PMC3917532; doi:10.1371/journal.pone.0087950)
Supplement: Text S1 — Additional details on the A. aeolicus metabolic network reconstruction and curation. (PDF) [file pone.0087950.s001.pdf]

Text S1

Introduction

Attached File S1 (A.aeolicus.xml) describes the whole-genome metabolic model of *A. aeolicus*, encoded in the SBML language [1]. To facilitate use and future work, the model is carefully organized by sub-system, with notes (highlighted with %% signs) added to each sub-system. An initial functional model was downloaded from the model SEED server [2], which then formed the basis for the subsequent curation process. The first steps in the curation process were to define the correct inputs (nutrient growth medium) and outputs (biomass) of the model. Before describing those steps, we first briefly outline Flux Balance Analysis (FBA), which was used to test the basic capability of the model to transform inputs to outputs. Finally, we highlight a few aspects of the gap-filling and curation process.

Flux balance analysis

The viability of the metabolic network of *A. aeolicus*, or its capacity to generate all biomass components from the nutrient inputs, was tested using Flux Balance Analysis (FBA). FBA has been described in detail elsewhere [3, 4], and relies on three basic equations:

$$\frac{dX_n}{dt} = \sum_m S_{mn}\nu_m \tag{1}$$

$$S \cdot \nu = 0 \tag{2}$$

$$Z = \sum_m c_m \nu_m \tag{3}$$

where X_n is the concentration of metabolite n, S_{mn} is the stoichiometry of metabolite n in reaction m, whose flux in turn is ν_m. The total metabolic network is thus described as an $n \times m$ matrix (S) of stoichiometries and an m-dimensional vector (ν) of fluxes. Under steady-state growth the network is required to obey the principles of mass balance, which is expressed as equation (2). Z is an objective function that is optimized, and consists of a linear combination of individual fluxes weighted by proportionality constants c_m. The objective function Z can be any output of choice, but in our case is the full set of biomass components of *A. aeolicus*. FBA was implemented in Matlab using the COBRA toolbox [5]

Growth medium and biomass composition

The total model includes a set of external metabolites that are imported into the model from a null vector using a set of exchange reactions. These external metabolites include both the nutrients that the network takes up (the growth medium) and the objective function as well as waste products that form the output of the network. Modifications of this external pool allows one to test the viability of the organism model in different growth conditions, or to modify the output biomass, for example to focus on different sub-systems as part of the curation process.

The initial model obtained from SEED was found to grow as a heterotroph on a range of dipeptides and other organics, while *A. aeolicus* is known to grow on inorganic nutrients only. To enforce autotrophy we therefore eliminated all organics from the growth medium, allowing only uptake of inorganic compounds. As outlined in the main text, *A. aeolicus* can use a range of inorganics for its energy metabolism, and as its sources for cellular nitrogen and sulfur. Thus, in addition to a set of trace minerals, we allowed environmental exchange of the model with the following compounds whose elements ("CHNOPS") make up the bulk of cellular material: H_2, O_2, H_2O, H^+, CO_2, HCO_3^-, NO_3^-, NO_2^-, NH_3, PO_4^-, S^0, H_2S, $S_2O_3^{-2}$, $S_4O_6^{-2}$, SO_4^{-2}.

The internal reactions of the network convert these inorganic nutrients into the components of biomass, including protein, DNA/RNA, membrane lipids, peptidoglycan and lipopolysaccharide. The relative composition of the biomass of *A. aeolicus* has not been as carefully evaluated as other organisms (such as *E. coli*) for which well-curated metabolic models exist. We therefore did not adjust the ratios of biomass components as defined in the initial model obtained from SEED, instead focusing more on getting the basic presence/absence of biomass components correct and ensuring that internal pathways of the model are capable of producing them. To use this model to accurately predict growth rate, systematic studies will be needed to quantify the ratios of the different components of biomass.

Many monomer biomass components (e.g. amino acids, nucleotides) are the same for all life forms, and most of our efforts in curating the biomass vector thus involved modifying the composition of the phospholipids, which in *A. aeolicus* are highly diverse and have distinct characteristics. Most of our modifications were based on numbers obtained from Ref. [6]. That study quantified in detail the lipids of *A. aeolicus*, and showed that in addition to the standard fatty acid ester lipids used by most bacteria, this species also contains both mono- and di-ether lipids. Table 1 shows the monomer fatty acid composition of *A. aeolicus* membranes. Numbers from Ref. [6] were adapted in the following manner to simplify representation: 1) Fatty acids with concentrations below 0.1% were excluded, 2) fatty acids with unsaturated bonds in the same position differing only in their stereo-conformation (cis/trans) were treated as equivalent, 3) abundances of di-ether lipids were distributed over their monomer components as part of a single composite di-ether lipid vector (see below), 4) abundances of fatty acids containing a cyclopropane ring were proportionally distributed over the unsaturated fatty acids from which they could be derived. This latter re-distribution was done because cyclopropane rings are generally a modification made to unsaturated fatty acids after their incorporation into the lipid membrane [7]. We leave the capture of cyclopropane ring generation (and most other such post-biosynthetic modifications of macromolecules) for future generations of the model.

Table S 1. Fatty acid (FA) composition of the *A. aeolicus* lipid membrane.

FA monomers*	Ester lipids	Mono-ether lipids	Di-ether lipids
14:0	0.001	0	0
16:0	0.010	0	0
16:1Δ9	0.001	0	0
16:1Δ7	0.004	0	0
18:0	0.141	0.729	0.604
18:1Δ11	0.029	0	0
18:1Δ9	0.045	0.022	0
19:0	0	0.002	0.013
20:0	0.034	0.103	0.158
20:1Δ13	0.084	0	0
20:1Δ11	0.647	0.139	0
21:0	0.001	0	0
21:1Δ13	0	0.005	0.225
22:1Δ13	0.003	0	0
Total	1.000	1.000	1.000
Membrane fraction	0.63	0.35	0.02

Notes: * Saturated FA's denoted as (:0), unsaturated as (:1), with Δ denoting the position of the double bond relative to the carboxyl end of the FA.

Model curation

In generating the putative functional metabolic models it lists online, the SEED server [2] performs automated gap-filling and curation. However, as noted above, the model we downloaded for *A. aeolicus*

was growing under a heterotrophic lifestyle, which imposes very different constraints on the curation (and thus gap-filling) procedure than a restriction to inorganic nutrients does. Moreover, as discussed in detail in the main text, we are interested primarily in the evolution of metabolic sub-systems, for which automated reconstruction is not desirable. Instead, we therefore manually evaluated each gap-fill required to allow basic autotrophic growth, as well as deciding which pathway represented the correct completion to a given components of biomass.

The sub-system with the single largest number of gap-fills was the pathway producing lipopolysaccharide. However, *A. aeolicus* does possess most of the genes for the standard pathway to Lipid A, all genes for the synthesis and attachment of KDO (keto-deoxyoctulosonate), and some of the genes for the synthesis of the heptose precursor for Core assembly [8]. Inner-outer core and O antigen assembly are not clear from the annotated genome of *A. aeolicus*, however, and we did't have a good basis for assessing which forms are synthesized. We therefore simply left these pathways as they were generated from the SEED server. Future experimental studies could help provide a solid empirical foundation for this part of the model.

Many other gap-fills were unambiguous because they involved completion of a pathway for which most (or all) other enzymes were present and no viable alternative pathways existed to complete a given sub-system. In a small number of cases we included gap-fills purely to ensure that the model was fully autotrophic with no organic compounds being exchanged with the environment. These gap-fills generally involved interconversion between compounds that could be easily explained through promiscuous catalysis by enzyme already being used for a number of similar reactions (for example purine-substitution interconversions). The lone exception is 5'-deoxyadenosine, which is produced in a number of cofactor biosynthetic pathways, and which is the single organic drain reaction in the model because we could find no plausible reaction or sequence to reincorporate it. A few other cases were more ambiguous, or of greater interest to us because they were part of a sub-system whose evolution we were investigating. In those case we relied on phylometabolic analysis (PMA) in the curation process, as detailed extensively in the main text.

We note one final aspect of the model curation as it relates to the fatty acid biosynthesis network. As detailed in the previous section here and in the main text, the membrane lipids of *A. aeolicus* are highly diverse, which led to a disproportionally large sub-network compared to other parts of the metabolism. To somewhat limit the ultimate size of this sub-network, while still capturing most of its reality, we used a few simplifications in the representation of the lipid component of the biomass vector, as outlined in the previous section. In addition, we used single composite reaction sequences to represent the synthesis of the di-acyl, mono-alkyl or di-alkyl glycerol moieties that make up the final assembled membrane. That is, we used reactions involving generic carboxylic acids (R-COOH) or alcohols (R-OH) for synthesis pathways leading to generic glyceroyl moieties, followed by one single reaction in which the generic R groups were replaced with acyl or alkyl groups according to the stoichiometry of Table 1. We chose this representation (fatty acid synthesis explicit, glyceroyl-group assembly implicit) because the key points of regulation in adapting lipid composition are when to terminate chain length extension, which substitution patterns to include and whether to transform the carboxylic acid head group into an alcohol group (for ether lipids) or not. The entire set of fatty acid, regardless of its composition, is then funneled into the final phospholipid assembly process.

References

1. Hucka M, Finney A, Sauro HM, Bolouri H, Doyle JC, et al. (2003) The systems biology markup language (SBML): a medium for representation and exchange of biochemical network models. Bioinformatics 19: 524–531.

2. Henry CS, DeJongh M, Best AA, Frybarger PM, Linsay B, et al. (2010) High-throughput generation, optimization and analysis of genome-scale metabolic models. Nature Biotechnology 28: 977–982.

3. Varma A, Palsson BO (1994) Metabolic flux balancing: Basic concepts, scientific and practical use. Bio/technology 12.

4. Kauffman KJ, Prakash P, Edwards JS (2003) Advances in flux balance analysis. Current opinion in biotechnology 14: 491–496.

5. Becker SA, Feist AM, Mo ML, Hannum G, Palsson BØ, et al. (2007) Quantitative prediction of cellular metabolism with constraint-based models: the COBRA Toolbox. Nature protocols 2: 727–738.

6. Jahnke LL, Eder W, Huber R, Hope JM, Hinrichs KU, et al. (2001) Signature lipids and stable carbon isotope analyses of octopus spring hyperthermophilic communities compared with those of aquificales representatives. Applied and Environmental Microbiology 67: 5179–5189.

7. Zhang YM, Rock CO (2008) Membrane lipid homeostasis in bacteria. Nature Reviews Microbiology 6: 222–233.

8. Schnaitman CA, Klena JD (1993) Genetics of lipopolysaccharide biosynthesis in enteric bacteria. Microbiological reviews 57: 655–682.
